# Overview of subjective cognitive decline in Brazil: a narrative review

**DOI:** 10.1590/1980-5764-DN-2025-0416

**Published:** 2026-06-01

**Authors:** Isabele Sessa Soares, Adalberto Studart-Neto, Sonia Maria Dozzi Brucki

**Affiliations:** 1 Universidade Federal do Estado do Rio de Janeiro, Escola de Medicina e Cirurgia, Rio de Janeiro RJ, Brazil.; 2 Universidade de São Paulo, Faculdade de Medicina, Hospital das Clínicas, São Paulo SP, Brazil.

**Keywords:** Cognition, Cognitive Dysfunction, Memory, Risk Factors, Dementia, Aging, Cognição, Disfunção Cognitiva, Memória, Fatores de Risco, Demência, Envelhecimento

## Abstract

Subjective cognitive decline (SCD) is characterized by the individual’s self-perception of cognitive decline, without functional impairment and objective confirmation through neuropsychological testing. SCD has been studied as a possible clinical marker of early stages of neurodegenerative diseases, especially Alzheimer’s disease. This article presents a narrative review of the available literature on the context of SCD in Brazil, covering conceptual aspects, diagnosis, etiologies, assessment measures used in the country, case outcomes, epidemiological data, and predictors of progression. Proposals for the evaluation and clinical monitoring of patients with SCD were also developed. Although there is growing scientific interest in the topic, gaps remain in the clinical approach to SCD, and a lack of national data and specific tools validated for the Brazilian population persists.

## RESUMO.

O Declínio Cognitivo Subjetivo (DCS) é caracterizado pela autopercepção de piora cognitiva por parte do indivíduo, sem que haja prejuízo funcional e confirmação objetiva em testes neuropsicológicos. O DCS tem sido estudado como um possível marcador clínico de estágios iniciais de doenças neurodegenerativas, em especial a Doença de Alzheimer (DA). O presente artigo é uma revisão narrativa da literatura disponível sobre o contexto do DCS no Brasil, abrangendo aspectos conceituais, realização de diagnóstico, etiologias, medidas avaliativas utilizadas no país, evolução de casos, dados epidemiológicos e preditores de progressão. Foram também elaboradas propostas para avaliação e acompanhamento clínico dos pacientes com DCS. Embora haja crescente interesse científico acerca do tema, ainda há lacunas na abordagem clínica do DCS e escassez de dados nacionais e de ferramentas específicas validadas para a população brasileira.

## INTRODUCTION

 Subjective cognitive decline (SCD) is among the most common complaints reported by older adults, which refers to an individual’s perception of worsening cognitive function without objective changes in formal neuropsychological assessments and without impairment in activities of daily living (ADLs)^1,2^. The diagnosis is based on the patient’s self-perception of cognitive decline compared to their previous state, normal performance on cognitive tests, and exclusion of neurological, psychiatric, or medical conditions that could explain the complaint^2, 3^. 

 Thus, it refers to the patient’s own understanding that their cognitive abilities are worsening over time, without the need for cognitive testing or third-party reports confirming the scenario^2^ . SCD is not related to a specific disease; however, studies indicate that this condition may precede mild cognitive impairment (MCI) and dementia^2,4^. 

 SCD has diverse etiologies. It can be caused by normal aging, depression, medical conditions, such as sleep disorders, and medication side effects. Studies show that SCD may also represent a preclinical stage of neurodegenerative diseases, such as Alzheimer’s disease (AD)^3,5^. Thus, SCD is now considered a condition of interest for both early screening and participant selection in clinical trials of preventive therapies. The global prevalence of SCD, according to two studies with large population samples, using around 30,000 individuals each, ranges from 23.8% to 46.4%^6,7^. However, in Brazil, evidence on the prevalence, validated diagnostic instruments, and longitudinal monitoring of SCD remains limited^4,8^. 

 This work aims to gather and analyze the main data available in the literature on SCD, with an emphasis on the Brazilian context, and to propose ways to improve the diagnostic and therapeutic approach for this population. 

## METHODS

**Figure 1:**
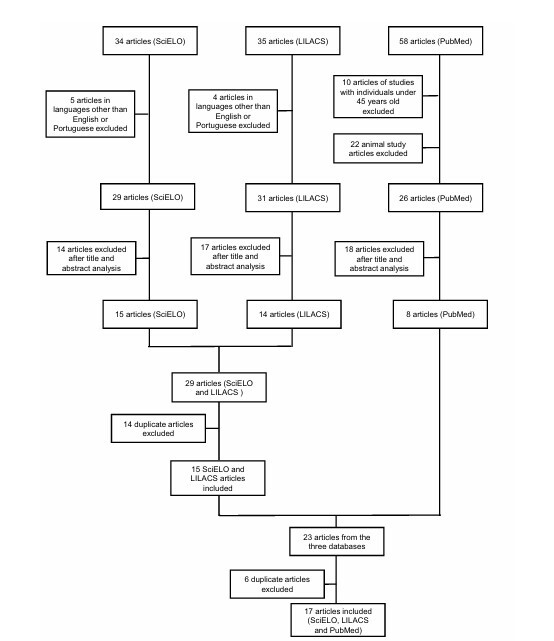
Flowchart of articles included from the baselines.

 The information for this narrative review was obtained from searches in the PubMed, SciELO, and LILACS databases (Figure 1). Articles were included according to the MeSH (Medical Subject Headings) term "Subjective Cognitive Decline." Articles were selected in English and Portuguese. Data collection period: from May 1982 to January 2026. The database searches identified a total of 127 articles related to SCD in Brazil, and 41 articles were excluded, primarily for not being in English or in Portuguese, involving animal studies or analyzing individuals under 45 years old. A total of 49 articles were excluded after title and abstract screening, leaving 37 studies. After removing 20 duplicates, 17 articles were included. An additional 44 articles were included from manual searches and articles used as references, provided they met the inclusion criteria: original articles, systematic reviews, and observational studies. 

### Overview

#### SCD diagnosis

 Identifying these patients is crucial for scientific advancement, especially in selecting candidates for clinical trials focused on preventative or disease-modifying treatments. In 2014, the international research group subjective cognitive decline-initiative (SCD-I) recommended two research criteria for SCD, with an emphasis on the study of preclinical AD. The first criterion is the patient’s perception, relative to their previous performance, of worsening cognitive abilities that are unrelated to an acute event. The second is the absence of significant deficits on standardized cognitive tests (adjusted for age, sex, and education). Furthermore, individuals who may have symptoms attributed to a psychiatric disorder, use medications or psychoactive substances, have neurological diseases, or are diagnosed with dementia or MCI do not qualify clinically for SCD^1-3^ . 

 To identify individuals most likely to develop MCI and dementia among those affected by SCD, the term ’SCD-plus’ was proposed. This concept defines patients most likely to develop neurodegenerative processes: those aged 60 or older and those who self-perceive progressive memory loss over the past 5 years, are concerned about this decline, and have family members who confirm the complaints^1 ,3^. In clinical practice, monitoring these individuals — especially those who meet the SCD-plus criteria — is essential for monitoring possible progression to dementia^1^ . 

### Etiologies

 SCD can have three different trajectories: reversible SCD, persistent SCD, or progressive SCD to dementia. In reversible SCD, the patient has cognitive complaints, but these may disappear, and the individual’s cognitive functions remain stable. That is, the condition is reversible and does not progress to objective cognitive decline. In this case, SCD is commonly associated with depression, sleep disorders, or the effects of medications with anticholinergic or GABAergic action^1,2^. Persistent SCD is characterized by irreversibility and the absence of progression to objective cognitive decline. This clinical profile may be caused by normal aging^2^ . Finally, progressive dementia-related SCD is marked by the development of MCI, later progressing to dementia. This progression can be caused by neurodegenerative diseases, such as AD^2,9^. 

### Evaluation measures in Brazil

 SCD, as characterized by an individual’s complaint without objective cognitive changes, cannot be detected in screening tests^1^ . Recent scientific literature highlights a wide variety of instruments and methodologies used to identify SCD and also points to significant methodological differences, especially regarding the cultural adaptation of these tools^4,8^. To investigate the evaluative measures of SCD in Brazil, eight individual studies^10-17^ and a systematic review^8^ comprising 25 studies^18-42^ were analyzed, totaling 32 studies (Table 1). 

**Table 1:** Summary of sample characteristics and evaluation results.

Study	Test	Population studied	Education (years), mean (SD)		Frequency of complaint individuals (%)	Male (%)		Female (%)		SCD group
			Total sample	Complaint group		Total sample (%)	Complaint group	Total sample (%)	Complaint group (%)	
Almeida^18^	Single question	220	—	—	59.1	30.4	28.50	69.6	71.5	No
Mattos et al.^19^	MAC-Q and single question	71	10.5	—	53.5	11	—	89	—	No
Argimon and Stein^20^	MAC-Q	66	2.54 (2.61)	—	—	23.9	—	76.1	—	No
Benites and Gomes^21^	MAC-Q and PRMQ	642	13,82 (3.02)	—	—	37	—	63	—	No
Minett et al.^22^	MAC-Q and questions to the patient	114	—	8.8	21	22.8	12	77.2	88	No
Caramelli and Beato^23^	MAC-Q	60	8.5 (5.5)	8.2 (5.6)	55	35	24.2	65	75.8	No
Lindôso^24^	MAC-Q	51	—	—	—	19.6	—	80.4	—	No
Lima-Silva and Yassuda^25^	MAC-Q and Forgetfulness frequency scale	57	—	—	—	12.3	—	87.7	—	No
Brucki and Nitrini^26^	Single question	163	0.83 (1.55)	0.7 (1.3)	69.9	49.7	43.9	50.3	56.1	No
Paulo and Yassuda^27^	MAC-Q	67	—	—	—	10.5	—	89.5	—	No
Aguiar et al.^28^	Single question	28	—	—	50	35.7	28.6	64.3	71.4	No
Kasai et al.^29^	SMC-scale	26	—	—	100	0	0	100	100	No
Piauilino et al.^30^	PRMQ	664	—	—	—	51.4	—	48.6	—	No
Santos et al.^31^	MAC-Q	204	—	8.5 (4.4)	49	23.5	24	76.5	76	No
Vale et al.^32^	MCS	161	4.6 (3.2)	—	—	41	—	59	—	No
Brum et al.^33^	MAC-Q and single question	56	—	—	—	35.7	—	64.3	—	No
Jacinto et al.^34^	Single question	248	—	—	59.3	—	—	---	—	No
Argimon et al.^35^	MAC-Q	121	3 (2.73)	—	—	31.4	—	68.6	—	No
Andrade and Novelli^36^	MAC-Q	90	—	—	71.11	4.4	—	95.5	—	No
Gil et al.^37^	Sunderland everyday memory questionnaire	79	—	—	100	24.1	—	75.9	—	No
Bourscheid et al.^38^	Single question	152	12.82 (4.23)	—	48.70	9.2	—	90.8	—	No
Bernardes et al.^39^	MAC-Q	386	—	—	35.7	4.1	—	95.9	—	No
Rizzi et al.^40^	Single question	45	—	11	26.7	28.9	25	71.1	75	Yes
Almeida et al.^41^	MCS (version A)	83	—	5.4 (4.0)	41	32.5	26.5	67.5	73.5	Yes
Dalpubel et al.^42^	MCS (version A)	100	5.12 (4.21)	5.4 (3.6)	45	32	24.4	68	75.6	No
Pereira et al.^11^	MAC-Q and single question	91	—	—	15	26.4	46.7	73.6	53.3	Yes
Studart-Neto et al.^15^	CFI	72	—	16.0 (5.0)	62.5	33.3	—	66.7	—	Yes
César-Freitas et al.^17^	Not specified	290	—	—	32.8	31.4	—	68.6	—	No
Borelli et al. (2022)^10^	Questions to the patient	6.687	—	4.8 (3.9)	37	47.5	43.1	52.5	56.9	Yes
César-Freitas et al.^13^	Question to the patient and the informant	215	—	—	40	—	—	—	—	Yes
Merlin and Brucki^14^	Not specified	65	12 (4.8)	—	30.77	35.38	—	64.62	—	Yes
Pereira et al.^10^	MAC-Q	57	5.49 (4.0)	—	31.6	19.3	—	80.7	—	Yes
Rochetti et al.^16^	MCS	71	—	14 (4.0)	23.9	—	35.3	—	64.7	Yes

Abbreviations: SD, Standard Deviation; MAC-Q, Memory Assessment Questionnaire; PRMQ, Prospective and Retrospective Memory Questionnaire; SMC-Scale, Subjective Memory Complaint Scale; MCS, Memory Complaint Scale; CFI, Cognitive Function Instrument; SCD, subjective cognitive decline.

 The systematic review examined questionnaires and questions used in scientific research in Brazil and Portugal to classify individuals with SCD. However, only data from Brazil were considered for this narrative review. It was not possible to identify adapted versions of the Sunderland Everyday Memory Questionnaire and Forgetfulness Frequency Scale used in the articles, which led to their exclusion from the critical analysis^8^ . Although the systematic review aims to analyze tools used to classify individuals with cognitive complaints, it is limited by the fact that most studies use assessment methods to identify only memory complaints. Only two studies^40,41^ used the classification of Subjective Cognitive Impairment, categorizing the individual’s impairment in cognition, not just memory. 

 In studies where the specific SCD condition was detected in the studied population^10-17,40,41^, largely based on the SCD criteria established by the SCD-I. In general, considering these studies, to exclude patients with objective cognitive decline, the main cognitive and functional screening tests used were the Mini-Mental State Examination (MMSE)^43^ , Brief Cognitive Screening Battery (BCSB)^44^ , and Functional Activities Questionnaire^45^ . The identification of cognitive complaints was mainly conducted by asking patients questions and using the Memory Assessment Questionnaire (MAC-Q)^46^ , with the most commonly used methodologies being the MAC-Q (15/33) and the question (12/33). Finally, the exclusion of individuals with anxiety and depression was done mainly using the Geriatric Depression Scale tool^47^ . 

 Although the MAC-Q questionnaire is frequently used, it has not been formally translated or validated for Portuguese speakers^8,46^. Furthermore, the test may not accurately identify individuals with SCD, as individuals may have concerns related to cognitive domains other than memory, such as attention and language, which cannot be screened by this test. 

 In the studies examined, one- or two-question assessments used phrases similar to "How is your memory?" and "Do you have memory problems?" This method is limited by the fact that small changes in the questions can be enough to generate significantly different results and that individuals’ responses can vary according to their level of education^8^ . 

 The Memory Complaint Scale (MCS) test emphasizes assessing the perception of memory problems. The tool has been formally validated and translated into Portuguese^8,32^. However, the systematic review highlights three limitations of this tool: the large number of abstract questions, the restricted validity analysis, and the absence of factor analysis. The exam stands out for having a questionnaire for the patient and another for the companion, a quality that is important for characterizing individuals with SCD-plus^8^ . 

 The application of Prospective and Retrospective Memory Questionnaire^30^ can be time-consuming due to the large number and length of questions. On the positive side, the questionnaire assesses short- and long-term prospective and retrospective memory. However, like the MAC-Q and MCS tests, it is also limited to investigating memory complaints. The instrument is valid for Portuguese speakers^8^ . 

 The cognitive function instrument (CFI), used to assess cognitive complaints in older adults without dementia, has been validated and translated into Brazilian Portuguese^15^ . Similar to the MCS, the CFI is an instrument that aids in classifying individuals with SCD-plus by obtaining responses from both the informant and the patient separately. The questions should be answered taking into account the individual’s cognitive status over the previous year. Furthermore, because it was initially designed to be conducted by email, a faceto-face interview is not necessary, and the patient can self-assess, which may be a limitation for its application to individuals with low educational levels^15,48 ,49^. 

 Thus, it is clear that the assessment methods (Table 2) primarily aim to classify memory complaints, with no significant analysis of cognitive concerns in a broader sense. This may compromise the identification of SCD, as the condition is not exclusively related to the self-perception of memory impairment. Furthermore, there is no standardized protocol in Brazil, so the choice of a particular test varies depending on the research objectives, the location where it is administered, and the patient’s level of education. Further studies are needed to validate a screening instrument that assesses SCD, including signs of SCD-plus, to increase its predictive value regarding the risk of objective cognitive decline. Therefore, the assessment tests used are considerably different, varying in approach, duration, number of items, and cognitive domains investigated^4,8^ . 

**Table 2:** Summary of the evaluation tests for subjective cognitive decline used in the articles.

Test	Number of items	Score minimum maximum	Observation	Cognitive domains assessed (number of items)	Strengths	Weaknesses	Number of studies using the test
MAC-Q	6	7–35 points	Self-reported questions with scores ranging from 1 to 5 points each, with the last question scoring twice. A score of 25 points or higher indicates memory problems	Episodic memory (5), attention (1) and language (1)	Short questionnaire	Without validation and translation into Portuguese	15
Single question	1	Objective question: "Yes" or "No"	Example: "Do you have memory problems?"	Episodic memory	Quick and easy-to apply assessment	Influenced by level of education, low reliability and no validation and translation into Portuguese	12
PRMQ	16	16–80 points	Self-reported questions scored from 1 to 5 points each. High scores reflect high self-reported memory problems	Episodic memory (16) and attention (5)	Validation and translation into Portuguese and evaluation of short term or long-term prospective and retrospective memory	Long application time	2
SMC-Scale	10	0–21 points	Questions with scores ranging from "No"=0 to "Yes"=1–3 points, with some scoring lower than others. The cutoff score is 3 points. A high score reflects a high rate of self-reported memory problems	Episodic memory (5), attention (2), language (2), orientation (2), processing speed (1)	Easy to understand	Without validation and translation into Portuguese	1
MCS	14 (7 for the patient and 7 for the companion)	0–14 points	Questions with scores ranging from 0 to 2 points each. Memory complaint: mild (3–6 points), moderate (7–10 points), severe (1–14 points)	Episodic memory (6), attention (1), activities of daily living (1)	Validation for the Portuguese language and questionnaire answered by the companion as well, useful for SCD-plus classification	Significant abstractness in the questions and validity analysis limited to internal consistency	3
CFI	14 (one version for the patient and the other for the companion)	0–14 points	Possible answers are "No"=0 , "Maybe"= 0.5, "Yes"=1 point and "Does not apply"	Memory, orientation, language and activities of daily living	Validation and translation for the Portuguese language and questionnaire answered by the companion as well, useful for SCD-plus classification	Influenced by the level of education and no consensus on the cutoff score	1

Abbreviations: MAC-Q, Memory Assessment Questionnaire; PRMQ, Prospective and Retrospective Memory Questionnaire; SMC-Scale, Subjective Memory Complaint Scale; MCS, Memory Complaint Scale; CFI, Cognitive Function Instrument; SCD, subjective cognitive decline.

### Evolution of patients with subjective cognitive decline

 Three regional longitudinal studies were analyzed to monitor the evolution of cognitive states in people aged 50 years and older. The study carried out in the city of Tremembé, in the state of São Paulo, revealed that among the 630 individuals analyzed, 365 did not present cognitive alterations, with this group being subdivided between those who reported cognitive complaints, that is, had SCD (27.6%, n=174), and those who did not report cognitive complaints (n=211)^17^ . After 5 years, 108 of the 174 individuals with SCD were reevaluated. Approximately 54% remained with the same diagnosis, with 12.9% progressing to MCI and 4.6% to dementia. One hundred and seven individuals, initially without complaints, were reevaluated, and 48% remained stable, 5.6% developed MCI, and 8.4% developed dementia^13^ . This research reinforces the idea that SCD may be a risk marker for MCI, since the percentage of individuals with SCD who progressed to MCI was approximately double the percentage of volunteers without cognitive impairment who developed MCI. However, the percentage of conversion to dementia in the group without cognitive complaints was slightly higher than that of the SCD group, demonstrating no significant association between SCD and dementia. The fact that 53.7% of participants remained diagnosed with SCD reinforces the stability of the condition and its relationship with non-neurodegenerative factors, such as depression and anxiety. There are some limitations, such as the small sample size and significant loss to follow-up. 

 In another longitudinal study, 102 individuals over 60 years of age who participated in the Brazilian Aging Memory Study program at the Hospital das Clínicas de São Paulo, conducted by the University of São Paulo, were evaluated and followed for 2 years. In the first evaluation, in 2017, 24.5% of the volunteers were classified as having no cognitive impairments or complaints, 24.5% as having SCD, and 51% as having MCI. Over the 2 years, 37 volunteers were lost to follow-up. In the second evaluation, among the 17 individuals initially classified as having no cognitive impairments who were reevaluated, 4 (23.5%) developed SCD; of those classified as SCD, 15.4% developed MCI. In the MCI group (n=35), five regressed to SCD, and three volunteers regressed to normal cognitive status14. SCD and MCI were characterized as unstable conditions, with SCD being considered a risk factor for objective, but not definitive, cognitive impairment. However, the small sample size and loss to follow-up (36%) compromise the statistical power of the study. 

 In the Brazilian longitudinal study carried out between 2016 and 2020, it involved 91 individuals with memory complaints, aged 50 years or older (mean of 67.6 years), and an average education level of 4–8 years, from a medium-sized city (Patos de Minas, in the state of Minas Gerais)^10,11^. In the first phase of the study, 15 (16.5%) participants were classified as having SCD, 45 (49.4%) as having MCI, and 31 (34.1%) as having dementia. After 4 years, 57 individuals were reevaluated; 50% converted from SCD to MCI, and 50% remained stable. Among the individuals initially classified as having MCI, 32 were reevaluated, and of these, 44% maintained the diagnosis, 34% regressed to SCD, and 19% progressed to dementia. Of the 31 individuals initially classified as having dementia, eight remained with dementia, seven regressed to MCI, and two to SCD. The improvement was due to treatment of reversible causes^10^ . Considering the total number of individuals initially diagnosed with SCD and evaluated in the final stage of the study, the conversion rate from SCD to MCI in the Patos de Minas study was the highest (50%). The statistical power of this study is compromised by the small sample size and loss to follow-up (37.3%). 

 A non-Brazilian systematic review of prospective cohort studies evaluated 8,128 individuals, separating them into a control group and a group with SCD. The study revealed that the SCD group was 2.15 times more likely to progress to MCI than the control group, while the chance of progressing to dementia was 2.17 times greater^50^ . 

 Thus, studies demonstrate the heterogeneity of SCD, which can be stable, transient, or progressive. This reinforces the need to monitor patients with cognitive complaints, even if they do not present subjective changes, and to investigate possible associated factors. These studies did not use the concept of SCD-plus, which would have a higher risk of conversion to MCI and dementia. 

### Epidemiology

#### Prevalence worldwide

 Two studies stand out for their large sample sizes. The Cohort Studies of Memory in an International Consortium (COSMIC) comprised 39,387 participants aged 60 and above with no cognitive impairment, encompassing 16 cohorts from 15 countries. The prevalence of SCD was 23.8% using qualitative standardization and 25.6% using item response theory. In this study, SCD was more common in men, people with low education, Asian and Black people, and residents of low- and middle-income countries^6^ . 

 The meta-analysis of the prevalence of SCD in China was based on 17 studies involving a total of 31,782 Chinese people aged over 60 years, with an SCD rate of 46.4% of the sample and a predominance in women (58.9%), thereby strengthening the association between female sex and SCD^7^ . 

 The significant difference in prevalence rates for SCD may be attributed to the varying methodologies employed in the studies. While the Chinese meta-analysis sampled only residents of a specific region of China, the COSMIC study encompasses individuals from 16 countries, providing a more representative sample group and reducing selection bias^6,7^. The COSMIC study utilized the MMSE to objectively classify individuals’ cognitive status, and in studies that did not use it, derived scores from similar tests to those used in the MMSE. Furthermore, it adopted the same criteria for identifying SCD in all cases and performed qualitative and quantitative harmonization of items from the SCD assessment methods used^6^ . However, in the research carried out in China, the different tests and criteria for diagnosing SCD were not standardized^7^ . The discrepancies observed in the identification of SCD resulting from the lack of standardization can influence the results obtained in studies, reinforcing the need for uniform diagnostic criteria for more accurate comparisons. 

### Prevalence in Brazil

 A study using data from the Brazilian Longitudinal Study of Older Adults (ELSI-Brazil) calculated the prevalence of SCD in Brazil at 29.21% (95% confidence interval [CI] 28.22–20.21). The study included a sample of 6,687 individuals aged 50 or older. This figure is significantly higher than that found in high-income countries, according to international comparative studies. Prevalence varied by region, sex, and age. Individuals classified as having cognitive decline were on average 62.6 (±9.4) years old, 56.9% were women, 52% were mixed race, and 70.7% had low levels of education^12^ . Women had higher rates of SCD than men, both in adults and in older adults, which may be related to the higher prevalence of depression and anxiety in this group^51^ . Older adults also had more cognitive complaints than adults aged 50 to 60, which is expected with advancing age. Some modifiable risk factors for dementia were associated with SCD, such as low education (30% illiterate), a sedentary lifestyle, and, especially, hearing loss, which was the factor most strongly linked to cognitive complaints^52^ . Furthermore, men with SCD had more hearing loss than women, something that was already analyzed in a cohort carried out in the United States of America and reinforces the need for early interventions^53^ . People with SCD also reported more depressive symptoms, feelings of loneliness, and poorer sleep quality, events already reported in other studies^54-56^. Self-reported race also showed an association with SCD: brown people presented more cognitive complaints, which may be related to social inequalities, limited access to healthcare, and greater vulnerability to disease. Even with limitations, such as the use of self-reported data and exclusion of participants due to missing data, the findings reinforce that SCD is common in Brazil and may indicate a future risk of dementia. Therefore, the study reinforces the importance of public prevention policies, with an emphasis on more vulnerable populations and primary health care. 

 No other large-scale population studies on the prevalence of SCD in Brazil were found. Furthermore, most scientific publications analyze objective rather than SCD. However, there are regional studies with small sample sizes. Three regional studies reporting the prevalence of SCD in participants were found, with prevalences of 16.5% and 27.6% in population-based studies and 24.5% in specialized memory clinics^12,14,17^. There are insufficient population studies in various regions with well-defined SCD criteria, which limits the generalization of available data and hinders the understanding of the early onset of SCD. 

### Predictors for subjective cognitive decline progression

 Although not all individuals progress to more severe conditions, certain predictive factors significantly increase the risk of progression. A systematic review identified 15 predictors of progression from SCD to objective cognitive decline. Four biomarkers were identified: Amyloid β_1-42_ (Aβ42) deposition, low Hulstaert Formula score, apolipoprotein E4, and hippocampal atrophy. Four epidemiological factors were identified: advanced age at baseline, depression, anxiety, and difficulty performing daily activities. Finally, seven neuropsychological factors were identified: advanced age at symptom onset, stable symptoms, worries, informant-confirmed cognitive decline, severe symptoms, and poor performance on the Trail Making Test B^57^ . 

 Another study indicated that low concentrations of cerebrospinal fluid (CSF) amyloid Aβ42 are a strong predictor of SCD conversion to AD, supporting the hypothesis that pathological events begin with Aβ42 deposition^4,58^. Aβ42 levels would be altered in the early stages of the disease, a period when the individual does not yet exhibit cognitive impairments. Meanwhile, high concentrations of Tau and P-Tau act as better predictors of the conversion of MCI to AD, that is, in more advanced stages of Alzheimer’s. However, high concentrations of Tau and P-Tau had less clinical significance in the study than low concentrations of Aβ42^58^ . 

 Through analysis of the available literature, we observed a scarcity of studies in Brazil, representing a gap that needs to be explored. However, three Brazilian studies^16,40,59^ were found that address the value of CSF biomarkers for differential diagnosis and prognosis in the preclinical stage of AD. 

 A 3-year follow-up research^40^ studied 45 individuals from a memory clinic in Southern Brazil and aimed to determine the accuracy of CSF Aβ42 and phosphorylated tau (p-Tau_181_) in the differentiation of amnestic Mild Cognitive Impairment (aMCI) from SCI. The study concludes that the level of Aβ42 was lower in the aMCI group than in the SCI group, while the concentration of p-Tau_181_ was similar in both groups. This result aligns with previous studies of AD biomarker articles cited by the study itself, in which the level of CSF Aβ42 decreases before p-Tau_181_ increases, and the concentration of p-Tau_181_ only rises significantly after advanced neurodegeneration. Therefore, at the time of collection, individuals with aMCI had not yet experienced the increase in levels of CSF p-Tau_181_, which makes prediction impossible. Therefore, the level of Aβ42 proved useful in discriminating between aMCI and SCI subjects, and the concentration of CSF p-Tau_181_ alone was insignificant in predicting aMCI diagnosis^40^ . The Brazilian study reinforces the role of Aβ42 as a marker in distinguishing early cognitive impairment from subjective cognitive complaints alone. 

 A new study^59^ was conducted based on the previously mentioned longitudinal follow-up^40^ of the aMCI group to identify whether Aβ42 and p-Tau_181_ levels could be predictors of aMCI progression to Alzheimer’s disease dementia (ADD). Of the 33 aMCI individuals, 31 were reassessed. A total of 38.7% of aMCI subjects progressed to ADD. The relative risk of a subject with CSF Aβ42 levels lower than 618.5 pg/mL developing ADD was 17.4 times higher than in those whose levels were higher than 618.5 pg/mL. The p-Tau_181_ concentration did not significantly predict progression. 

 The third study16 aimed to relate neuropsychiatric symptoms with Aβ42 deposition in individuals with SCD and MCI. A negative correlation was found between scores on domain C of the Mil Behavioral Impairment Checklist (MBI-C), the Impulse Dyscontrol domain, and Aβ42 concentration, suggesting that amyloid pathology may be related to symptoms of impulsivity, agitation, aggression and disinhibition in pre-dementia stages. There was no correlation with p-Tau_181_ levels, which may indicate that the individuals evaluated were in a very early stage of the disease. The research provides evidence that behavioral changes may be an initial manifestation of AD. 

### Proposal for monitoring patients with subjective cognitive decline

**Figure 2:**
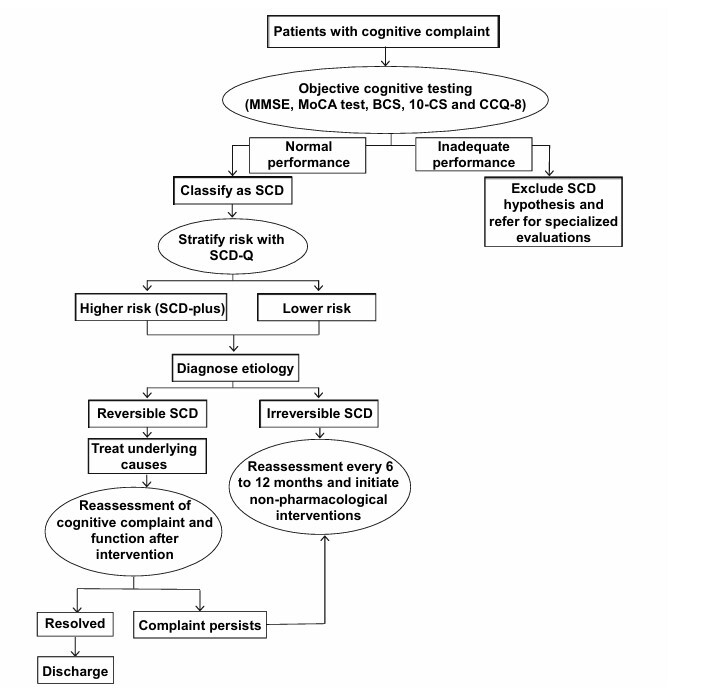
Clinical flowchart for the evaluation and follow-up of subjective cognitive decline individuals.

 The proposed approach is to identify SCD using a structured protocol based on the criteria established by the international SCD-I working group, adapted to the context of Brazilian Primary Health Care (Figure 2). Individuals with SCD are those who report self-reported cognitive complaints, exhibit no functional impairment, and demonstrate normal performance on objective cognitive tests. 

 Individuals with cognitive complaints should first undergo objective cognitive testing, so that patients with inadequate performance are excluded from the hypothesis of SCD and referred for specialized evaluations, while patients with expected performance but who continue to complain are classified as having SCD. In Brazil, the suggested brief cognitive assessment includes the MMSE, MoCA test, and the BCSB^1^ . Another instrument that can be included in the assessment is the 10-CS (10-point Cognitive Screener)^60^ , associated with the Cognitive Change Questionnaire (CCQ-8)^61^ , both of which are recommended by the Ministry of Health for use in primary care. When there are doubts or uncertainties regarding cognitive status, SCD should be assessed preferably through a comprehensive neuropsychological evaluation. 

 After identifying patients with SCD, those at higher risk of progression to MCI and dementia, such as individuals with SCD-plus, should be investigated. The internationally validated Subjective Cognitive Decline Questionnaire (SCD-Q) enables standardization of complaints, distinguishing nonspecific changes from conditions with a higher risk of conversion to MCI. 

 Furthermore, it is necessary to diagnose the etiology of the condition, distinguishing between reversible, persistent, and progressive SCD. Those with non-reversible SCD should be monitored and reassessed periodically (every 6–12 months), including reapplication of subjective scales and cognitive screening. Although specific pharmacological treatments for SCD are not yet used, non-pharmacological interventions have been studied to prevent progression to MCI and dementia. Strategies studied include cognitive training, regular physical activity, and nutritional counseling^1^ . 

 Therefore, the proposal links the qualification of SCD diagnosis with standardized instruments, structured longitudinal follow-up with periodic clinical and cognitive reassessments, and the implementation of non-pharmacological interventions. This can improve the quality of life for individuals with cognitive complaints and prevent progression to objective cognitive decline. 

 In conclusion, SCD is a heterogeneous condition, with distinct possible trajectories and etiologies, ranging from reversible causes — such as depression, sleep disorders, and medication use — to early manifestations of neurodegenerative pathologies^1,2,9^. Studies also indicate that people with SCD-plus characteristics have a higher risk of progression to MCI and dementia. 

 However, Brazilian data remain scarce, with few population-based studies and few screening instruments adapted to the Brazilian population. The assessment methods used in Brazil predominantly focus on evaluating memory complaints rather than the individual’s perception of their overall cognitive abilities. This prevents the standardization of diagnoses based on the SCD-I group criteria. Most studies on the epidemiology of SCD in Brazil are regional, use small population samples, and focus on the progression of the patient’s clinical condition. Still, the prevalence of SCD in Brazil has been shown to be in the range of 16.5–29.2%, being more frequent in women, people with low education, and the elderly. This percentage of individuals with SCD is significant and must be considered a public health issue, requiring screening, monitoring, and intervention in SCD cases to mitigate the impact on the quality of life of these citizens. 

 Despite this, longitudinal studies suggest that SCD may be an early marker of objective, though not deterministic, cognitive decline risk. Furthermore, standardized clinical monitoring protocols and tools specific to the primary care setting are scarce. Therefore, it is proposed that cognitive and functional assessment, along with follow-up plans and non-pharmacological interventions, be integrated into routine primary care in Brazil. This approach may favor more effective management of SCD and the prevention of objective cognitive decline in the Brazilian population. 

## Data Availability

The datasets generated and/or analyzed during the current study are available from the corresponding author upon reasonable request.
